# Distinct roles of ADIPOR1 and ADIPOR2: A pan-cancer analysis

**DOI:** 10.3389/fendo.2023.1119534

**Published:** 2023-02-21

**Authors:** Zhuoyuan Chen, Huiqin Yang, Yunfeng Ren, Ze Yang, Jiazheng Huang, Cheng Li, Ying Xiong, Bin Yu

**Affiliations:** ^1^ Central Laboratory of Yan’an Hospital Affiliated to Kunming Medical University, Kunming, Yunnan, China; ^2^ Department of Orthopedics of Yan’an Hospital Affiliated to Kunming Medical University, Kunming, Yunnan, China; ^3^ Greehey Children’s Cancer Research Institute, University of Texas Health at San Antonio, San Antonio, TX, United States

**Keywords:** adipose tissue, endocrine, AdipoR1, AdipoR2, tumor immune microenvironment, immunotherapy

## Abstract

**Introduction:**

AdipoR1 and AdipoR2 proteins, encoded by ADIPOR1 and ADIPOR2 genes respectively, are the receptors of adiponectin secrected by adipose tissue. Increasing studies have identified the vital role of adipose tissue in various diseases, including cancers. Hence, there is an urgent need to explore the roles of AdipoR1 and AdipoR2 in cancers.

**Methods:**

We conducted a comprehensive pan-cancer analysis for the roles of AdipoR1 and AdipoR2 via several public databases, including expression differences, prognostic value, and the correlations with tumor microenvironment, epigenetic modification, and drug sensitivity.

**Results:**

Both ADIPOR1 and ADIPOR2 genes are dysregulated in most cancers, but their genomic alteration frequencies are low. In addition, they are also correlated with the prognosis of some cancers. Although they are not strongly correlated with tumor mutation burden (TMB) or microsatellite instability (MSI), ADIPOR1/2 genes display a significant association with cancer stemness, tumor immune microenvironment, immune checkpoint genes (especially CD274 and NRP1), and drug sensitivity.

**Discussion:**

ADIPOR1 and ADIPOR2 play critical roles in diverse cancers, and it is a potential strategy to treat tumors through targeting ADIPOR1 and ADIPOR2.

## Introduction

Obesity is a significant contributor to the risk of cardiovascular disease, diabetes, and cancers. With the obesity epidemic, adipose tissue attracts more and more attention. Adipose tissue is an organ with a variety of roles in biological activity, including energy storage, thermogenesis, as well as endocrine. As an endocrine organ, adipose tissue is capable of secreting several hormones, such as leptin, fibroblast growth factor-21, interleukin-6, and adiponectin (1). Adiponectin is a peptide that predominately exists in the endocrine factors from adipocytes (2). After posttranslational modification, adiponectin is released into circulation and then binds to AdipoR1 and AdipoR2 (encoded by ADIPOR1 and ADIPOR2, respectively), which consequently initiates a range of downstream signal pathways (3).

A number of diseases have been linked to ADIPOR1 and ADIPOR2. Insulin resistance associated with obesity is accompanied by the downregulation of ADIPOR1 and ADIPOR2 (4). Adiponectin signaling is able to inhibit liver fibrosis and AdipoR2 is the main adiponectin receptor responsible for anti-fibrosis effect (5). Besides, ADIPOR1 is essential for vision, and its deletion causes photoreceptor dysfunction in mice model (6). AdipoR1 and AdipoR2 also play distinctive functions. They are required for the maintenance of membrane fluidity in most human cell types, which is independent of adiponectin (7). The dysregulation of ADIPOR1/2 is observed in various cancers. A study reported that both ADIPOR1 and ADIPOR2 are highly increased in gastric cancer samples from patients in Iran (8). Adrenal cancers are also associated with ADIPOR1 and ADIPOR2. Cortical cancer and pheochromocytomas had considerably higher levels of ADIPOR1 and ADIPOR2 as well (9). In addition, increasing studies have showed that the functions of immune cells are significantly influenced by AdipoR1 and AdipoR2. It has been determined that a subpopulation of Treg cells that secret more IL-10 express AdipoR1 (10). AdipoR1 is also able to induce the differentiation of naïve T cell into Th17 cells, and its deletion downregulates the expression of a series of T cell related genes (11). AdipoR1 and AdiporR2 are also found to be enriched in the dendritic cells (DCs) from patients with metastatic or locally advanced breast cancer. The anti-tumor impact of DCs can be diminished by AdipoR1 activating the AMPK and MAPKp38 pathways and AdipoR2 initiating the COX-2 and PPAR pathways (12).

In summary, ADIPOR1 and ADIPOR2 display the capacity to influence anti-cancer immunity and play vital roles in numerous tumors. However, there is no study to investigate their roles in pan-cancer. Hence, using a number of public databases, we carried out this study to explore the roles of ADIPOR1 and ADIPOR2 across cancers.

## Materials and methods

### The expression of ADIPOR1 and ADIPOR2

Firstly, the RNA expression levels of ADIPOR1 and ADIPOR2 in normal tissues were explored through The Human Protein Atlas database (THPA, https://www.proteinatlas.org/). Secondly, the RNA expression levels of ADIPOR1 and ADIPOR2 in tumor tissues were analyzed using TIMER2.0 database (http://timer.cistrome.org/) and GEPIA2 database (http://gepia2.cancer-pku.cn/#index). TIMER2.0 provides a web tool to perform biological analysis based on the data from TCGA database. GEPIA2 is a visualization website to conduct biological analysis based on the data from TCGA and GTEx database. Tumor acronyms were shown at the “Abbreviation” section. Thirdly, the protein levels of ADIPOR1 and ADIPOR2 were analyzed using UALCAN database (http://ualcan.path.uab.edu/).

### The prognostic value of ADIPOR1 and ADIPOR2

The prognostic value of ADIPOR1 and ADIPOR2 were estimated by UCSCXenaShiny and GEPIA2. UCSCXenaShiny is a R shiny application that provides an interactive tool to analysis data from TCGA, GETx, CCLE, and PCAWG, using which we explored the prognostic roles of ADIPOR1 and ADIPOR2 *via* univariate Cox analysis. In the GEPIA2 web, “Survival Map” module was use to draw the prognosis-associated heatmap, and “Survival Analysis” was used to draw the Kaplan-Meier plots (KM plots).

### The genetic alteration and epigenetic modulation

We explored the genomic alteration of ADIPOR1/2 in cBioPortal database (https://www.cbioportal.org/). The TCGA pan-cancer atlas studies were chosen as data source. The DNA methylation level of ADIPOR1/2 were analyzed *via* UCSCXenaShiny. Finally, the correlation of ADIPOR1/2 with DNA methyltransferases and N^6^-methyladenosine (m6A) enzymes were analyzed through TIMER2.0 database.

### Immune infiltration analysis

The expression of ADIPOR1/2 in normal immune cells were explored through THPA database. The immune infiltration cells across cancers were estimated through TIMER2.0 database. EPIC was chosen as the calculating method. Spearman test was chosen to estimate the correlation between immune cells and ADIPOR1/2. The roles of ADIPOR1/2 in immune subtypes were analyzed in TISDIB database (http://cis.hku.hk/TISIDB/).

### Stemness, TMB, MSI, and immunotherapy

The correlation between ADIPOR1/2 and stemness, tumor mutation burden (TMB), and microsatellite instability (MSI) were estimated *via* UCSCXenaShiny. The roles of ADIPOR1/2 in immunotherapy were analyzed through TIGER database (http://tiger.canceromics.org/#/), which provides a web-accessible tool to analyze the gene expression data correlated with immunotherapy. Finally, the correlation of ADIPOR1/2 with immune checkpoint genes across cancers was estimated through TIMER2.0 database.

### Function analysis

Firstly, we collected ADIPOR1/2 related genes from several public databases. We got 20 genes from geneMANIA database (https://genemania.org/), 20 genes from STRING database (https://string-db.org/), and 100 genes from GEPIA2 database. Totally, 138 ADIPOR1-related genes and 134 ADIPOR2-related genes were collected. Then, based on these genes, we explored the function of ADIPOR1/2 through Metascape database (https://metascape.org/gp/index.html#/main/step1). Thirdly, we analyzed the role of ADIPOR1/2 in cancers *via* CancerSEA database (http://biocc.hrbmu.edu.cn/CancerSEA/). CancerSEA provides a web tool for the exploration of genes across cancers at single cell level (13). Finally, based on the Pan-cancer data from UCSCXena database, the correlations between hallmark pathways and ADIPOR1/2 were calculated by spearman test.

### Drug sensitivity

CellMiner is a query tool for the genetic and drug sensitivity data of NCI-60 cancer cell lines, based on which we made an investigation into the correlations between ADIPOR1/2 and drug sensitivity.

### Statistical analysis

This study was conducted by several public database. Spearman test was used to estimate the correlations of ADIPOR1/2 with molecular features.

## Result

### The expression of ADIPOR1 and ADIPOR2

**Figure 1:**
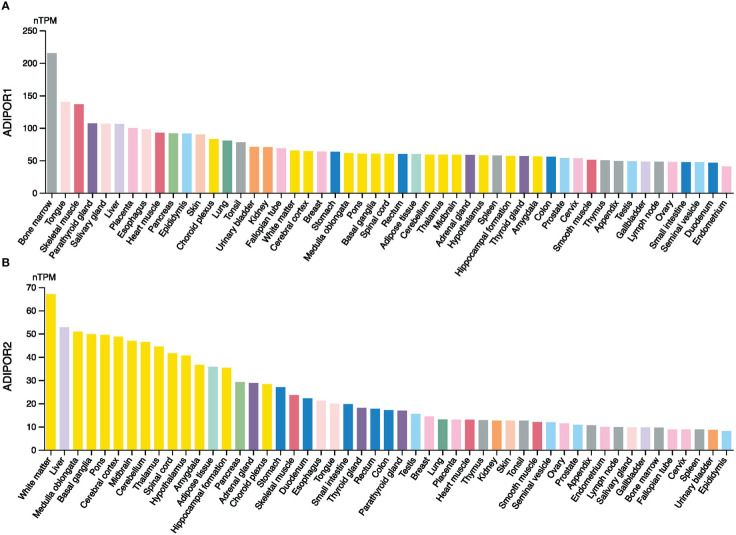
The expression levels of ADIPOR1 **(A)** and ADIPOR2 **(B)** in various normal tissues.

The RNA expression levels of ADIPOR1 and ADIPOR2 in normal tissues were exhibited in **Figure 1**. Generally, ADIPOR1 had a higher expression level in normal tissues then ADIPOR2. Bone marrow was the tissue with highest expression of ADIPOR1, followed by tongue and skeletal muscle (**Figure 1A**). White matter was the tissue with highest expression of ADIPOR2, followed by liver and medulla oblongata (**Figure 1B**).

**Figure 2:**
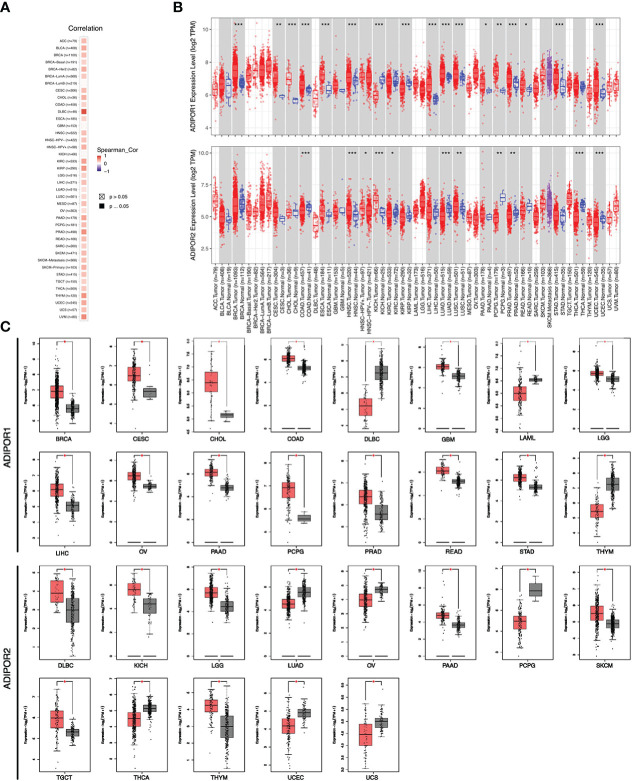
The expression of ADIPOR1 and ADIPOR2 across cancers. **(A)** The correlation between ADIPOR1 and ADIPOR2 across cancers. **(B)** The difference analysis ofADIPOR1 and ADIPOR2 across cancers *via* TIMER2.0 database. Red represented tumor samples, and black represented normal samples. *P < 0.05, **P< 0.001, ***P < 0.0001. **(C)** The difference analysis of ADIPOR1 and ADIPOR2 across cancers *via* GEPIA2 database. *P < 0.05.

ADIPOR1 and ADIPOR2 exhibited a wide positive correlation across cancers (**Figure 2A**). The results from TIMER2.0 database revealed that ADIPOR1 was upregulated in breast invasive carcinoma (BRCA), cervical squamous cell carcinoma and endocervical (CESO), cholangiocarcinoma (CHOL), colon adenocarcinoma (COAD), esophageal carcinoma (ESCA), head and neck squamous cell carcinoma (HNSC), liver hepatocellular carcinoma (LIHC), lung adenocarcinoma (LUAD), lung squamous cell carcinoma (LUSC), pancreatic adenocarcinoma (PAAD), pheochromocytoma and paraganglioma (PCPG), prostate adenocarcinoma (PRAD), rectum adenocarcinoma (READ), stomach adenocarcinoma (STAD), and uterine corpus endometrial carcinoma (UCEC), but was downregulated in kidney Chromophobe (KICH), and kidney renal papillary cell carcinoma (KIRP); ADIPOR2 was upregulated in HNSC, KICH, and LUSC, but was downregulated in COAD, kidney renal clear cell carcinoma (KIRC), LUAD, PCPG, PRAD, thyroid carcinoma (THCA), and UCEC (**Figure 2B**). Additionally, ADIPOR2 was upregulated in HNSC-human papillomavirus (HPV) (+) samples compared with HNSC-HPV (-) samples. The results from GEPIA2 database showed that ADIPOR1 was upregulated in BRCA, CESC, CHOL, COAD, glioblastoma multiforme (GBM), brain lower grade glioma (LGG), LIHC, ovarian serous cystadenocarcinoma (OV), PAAD, PCPG, PRAD, READ, and STAD, but was downregulated in lymphoid neoplasm diffuse large B-cell lymphoma (DLBC), acute myeloid leukemia (LAML), and thymoma (THYM); ADIPOR2 was upregulated in DLBC, KICH, LGG, PAAD, skin cutaneous melanoma (SKCM), testicular germ cell tumors (TGCT), and THYM, but was downregulated in LUAD, OV, PCPG, THCA, UCEC, and uterine carcinosarcoma (UCS) (**Figure 2C**).

**Figure 3:**
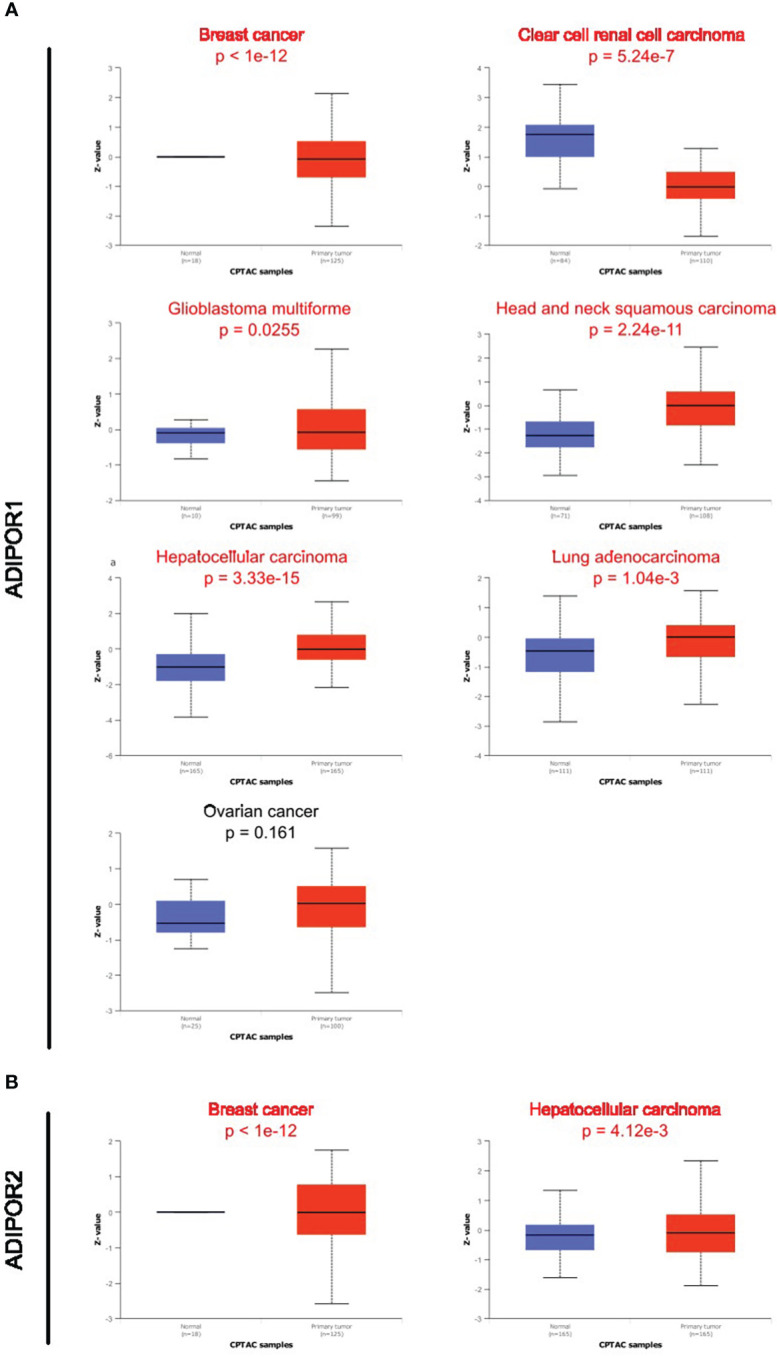
The protein abundance of ADIPOR1 and ADIPOR2 in cancers. **(A)** The ADIPOR1 protein abundance in UALCAN database. **(B)** The ADIPOR2 protein abundance in UALCAN database.

Data on ADIPOR1/2 proteins in cancer tissues were supplied from the UALCAN database. ADIPOR1 protein was dysregulated in breast cancer, clear cell renal cell carcinoma, glioblastoma multiforme, head and neck squamous carcinoma, hepatocellular carcinoma, and lung adenocarcinoma (**Figure 3A**). ADIPOR2 protein was dysregulated in breast cancer and hepatocellular carcinoma (**Figure 3B**).

### The prognostic value of ADIPOR1 and ADIPOR2

**Figure 4:**
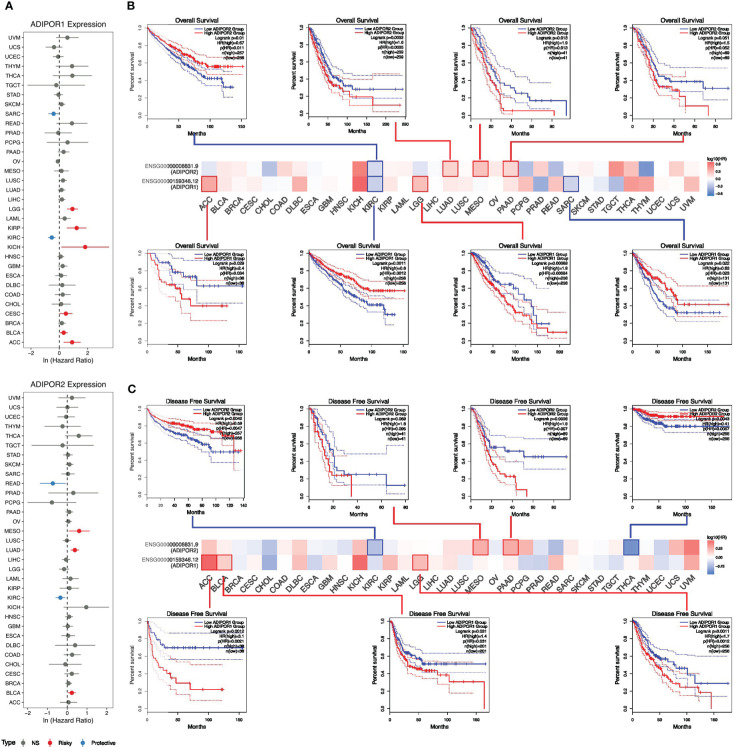
The roles of ADIPOR1 and ADIPOR2 in cancers. **(A)** The univariate cox analysis for ADIPOR1 and ADIPOR2. **(B)** The overall survival analysis for ADIPOR1 and ADIPOR2. **(C)** The disease free survival analysis for ADIPOR1 and ADIPOR2.

The results of univariate cox analysis revealed that ADIPOR1 worked as a risky factor in adrenocortical carcinoma (ACC), bladder urothelial carcinoma (BLCA), CESC, KICH, KIRP, and LGG, but worked as a favorable factor in KIRC and sarcoma (SARC); ADIPOR2 served as a risky factor in BLCA, LUAD, and mesothelioma (MESO), but served as favorable factor in KIRC and READ (**Figure 4A**). The survival analysis from GEPIA2 provided similar results. **Figure 4B** exhibited the overall survival (OS) analysis results of ADIPOR1/2 across cancers. The higher expression of ADIPOR1 was correlated with ACC and LGG, but was correlated with better OS in KIRC and SARC. The higher expression of ADIPOR2 was correlated with worse OS in LUAD, MESO, and PAAD, but was correlated with better OS in KIRC. **Figure 4C** showed the results of disease free survival (DFS) analysis. The higher expression of ADIPOR1 was correlated worse DFS in ACC, BLCA, and LGG. The higher expression of ADIPOR2 was correlated with worse DFS in MESO and PAAD, but was correlated with better DFS in KIRC and THCA.

### Genetic alteration and epigenetic modulation

**Figure 5:**
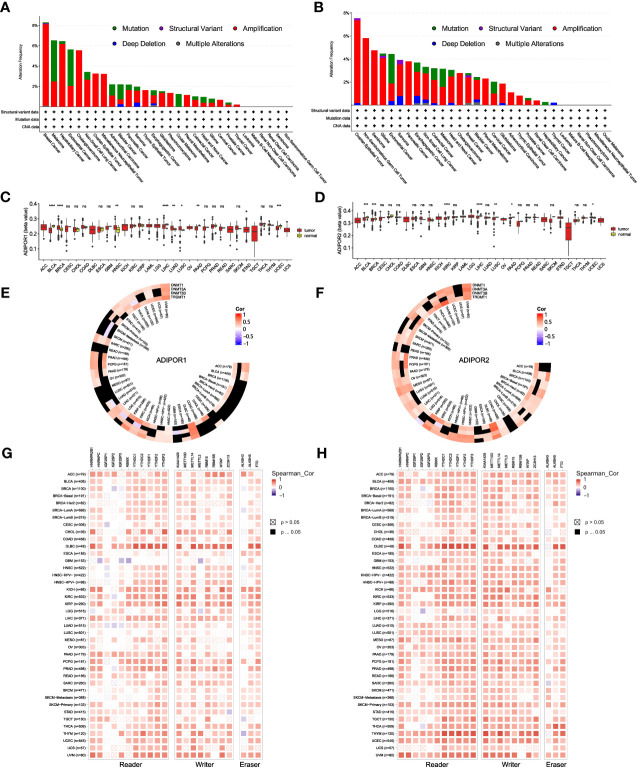
The genetic alteration and epigenetic modulation of ADIPOR1 and ADIPOR2. **(A, B)** The genetic alteration of ADIPOR1 and ADIPOR2 across cancers. **(C, D)** The DNA methylation of ADIPOR1 and ADIPOR2. **(E, F)** The correlation between ADIPOR1/2 and DNA methyltransferases. Black represented the relationship without significance. **(G, H)** The correlation between ADIPOR1/2 and m6A enzymes. *P < 0.05, **P < 0.01, ***P < 0.001, ****P < 0.0001 and ns presented P > 0.05.

Low genetic alteration frequency was seen for both ADIPOR1 and ADIPOR2 across cancers (**Figures 5A, B**). Amplification is the most common alteration of ADIPOR1/2. The tumor with the highest frequency of ADIPOR1 alteration is breast cancer, while the tumor with the highest frequency of ADIPOR2 alteration is ovarian epithelial tumor. **Figures 5C, D** exhibited the DNA methylation levels of ADIPOR1/2 across cancers, respectively. The DNA methylation level of ADIPOR1 was downregulated in BLCA, LIHC, PAAD, and UCEC, but was upregulated in BRCA, HNSC, LUAD, and LUSC. The DNA methylation level of ADIPOR2 was downregulated in BLCA, BRCA, KIRC, LIHC, LUSC, and PAAD, but was upregulated in UCEC. ADIPOR1/2 also exhibited a wide correlation with DNA methyltransferases (**Figures 5E, F**) and m6A enzymes (**Figures 5G, H**).

### Immune infiltration analysis

**Figure 6:**
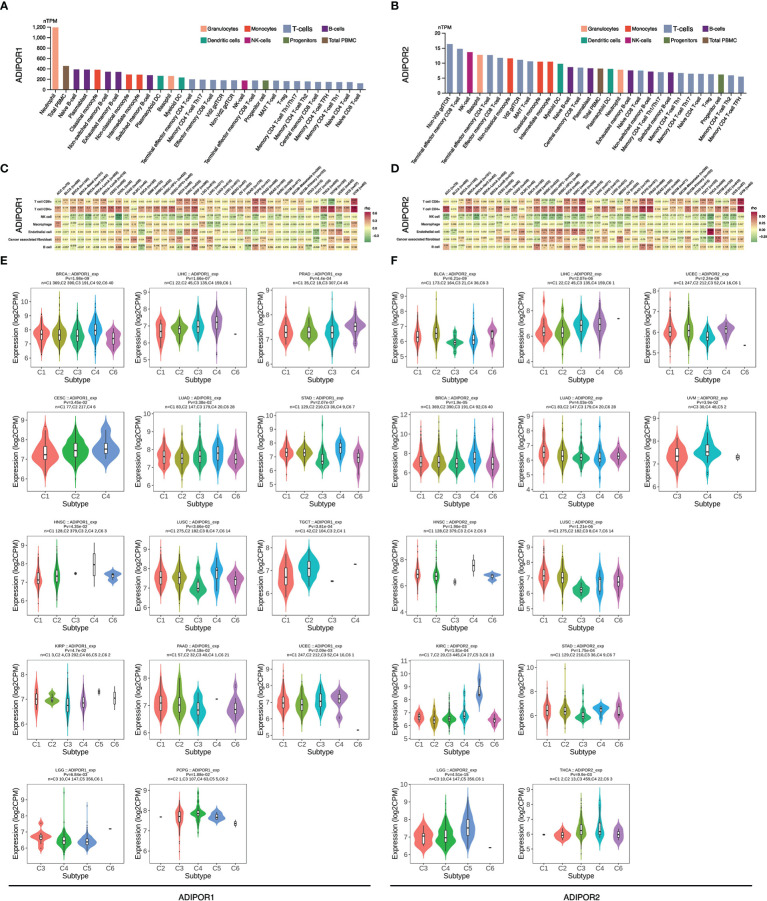
The correlation of ADIPOR1/2 with tumor immune microenvironment. **(A, B)** The expression of ADIPOR1/2 in immune cells. **(C, D)** The correlation between ADIPOR1/2 and tumor microenvironment. **(E, F)** The distribution of ADIPOR1/2 in immune subtypes of cancers.

The results from THPA database showed that neutrophil was the immune cell with the highest expression of ADIPOR1 (**Figure 6A**), and Non-Vd2 gdTCR was the immune cell with the highest expression of ADIPOR2 (**Figure 6B**). According to the EPIC findings, ADIPOR1 and ADIPOR2 were positively correlated with CD8+ T cell and negatively correlated with NK cells widely (**Figures 6C, D**). ADIPOR1 was correlated with immune subtypes in 17 cancers (**Figure 6E**). In general, subtype C4 had higher ADIPOR1 expression levels. ADIPOR2 was correlated with immune subtypes in 12 cancers (**Figure 6F**). ADIPOR2 was found to be mostly strongly expressed in subtype C4 and C5.

### Stemness, TMB, MSI, and immunotherapy

**Figure 7:**
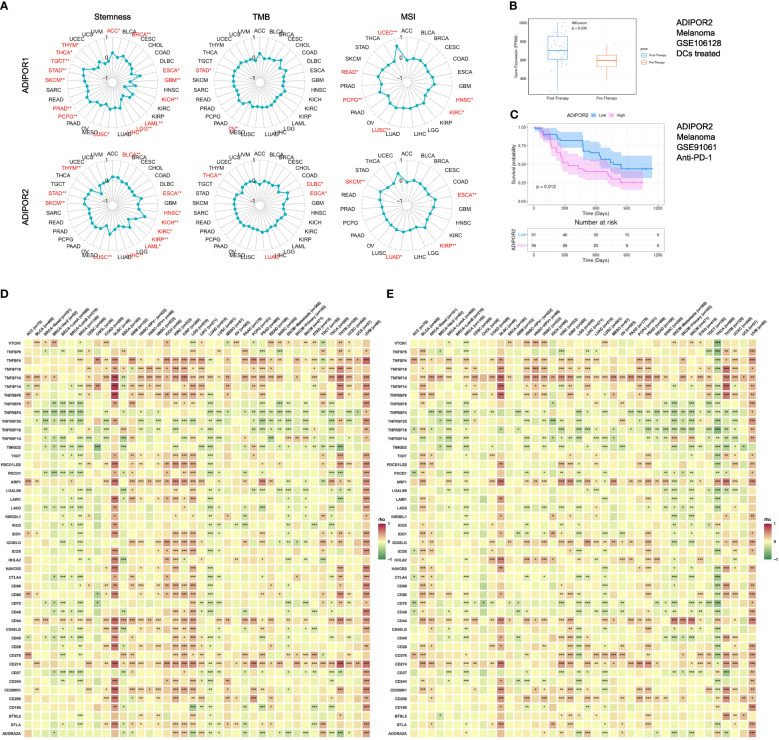
The correlation between ADIPOR1/2 and stemness, TMB, MSI, immunotherapy, and immune checkpoint genes. **(A)** The correlation between ADIPOR1/2 and stemness, TMB, and MSI across cancers. **(B)** The expression level of ADIPOR2 between Pre-Therapy and Post-Therapy. **(C)** The Kaplan-Meier plot of ADIPOR2 in immunotherapy cohort. **(D, E)** The correlation between ADIPOR1/2 and immune checkpoint genes. *P < 0.05, **P < 0.01, ***P < 0.001.

ADIPOR1 was correlated with stemness in ACC, BRCA, ESCA, GBM, KICH, LAML, LGG, LIHC, LUSC, PCPG, PRAD, SKCM, STAD, TGCT, THCA, and THYM, TMB in OV and STAD, and MSI in HNSC, KIRC, LUSC, PCPG, READ, and UCEC. ADIPOR2 was correlated with stemness in BLCA, ESCA, HNSC, KICH, KIRC, KIRP, LAML, LIHC, LUSC, SKCM, STAD, and THYM, TMB in DLBC, ESCA, LUAD, and THCA, and MSI in ESCA, KIRP, LUAD, and SKCM (**Figure 7A**). The results from TIGER database showed that ADIPOR2 was upregulated after DCs treated in GSE10618 cohort (**Figure 7B**), and the patients with low expression of ADIPOR2 had better clinical outcomes in GSE91061 anti-PD-1 cohort (**Figure 7C**). However, ADIPOR1 did not exhibited correlation with response to immunotherapy in clinical cohorts. Both ADIPOR1 and ADIPOR2 exhibited correlations with diverse immune checkpoint genes in various cancers. ADIPOR1 was predominantly positively correlated with TNSF4, TNFSF18, TNFSF15, TNFSF14, NRP1, CD44, CD276, and CD274, but was predominantly negatively correlated with TNFRSF4, TNFRSF25, TNFRSF18, TNFRSF14, and TMIGD2 (**Figure 7D**). ADIPOR2 was mainly positively correlated with TNFSF15, NRP1, ICOSLG, HHLA2, CD44, CD274, and CD200, but was mainly negatively correlated with TNFRSF4, TNFRSF25, TNFRSF14, TMIGD2, CD70, and CD48 (**Figure 7E**).

### Function analysis

**Figure 8:**
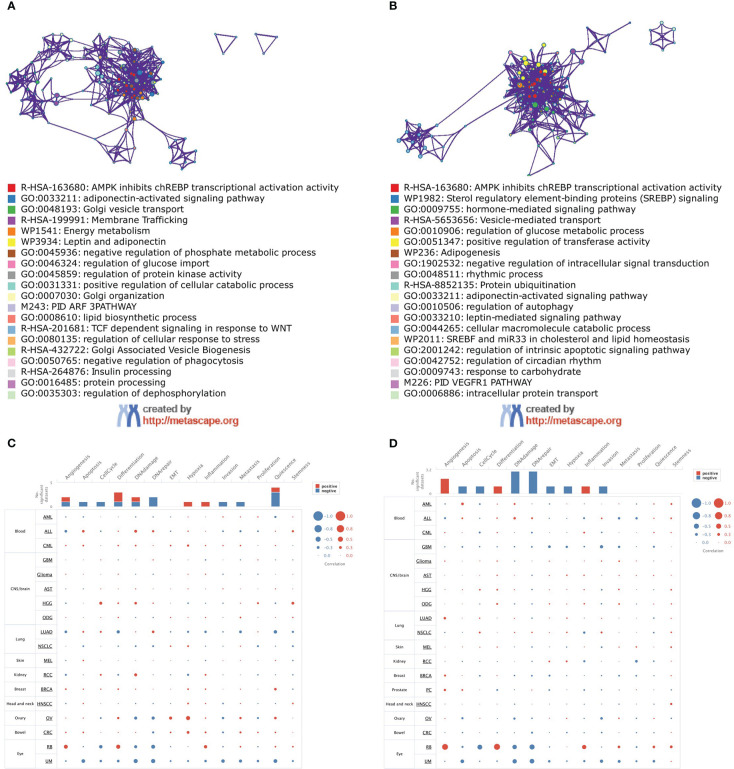
Function analysis of ADIPOR1 and ADIPOR2. **(A, B)** The function enrichment analysis of ADIPOR1/2 using MetaScape database. **(C, D)** The function analysis of ADIPOR1/2 using CancerSEA database.

**Figure 9:**
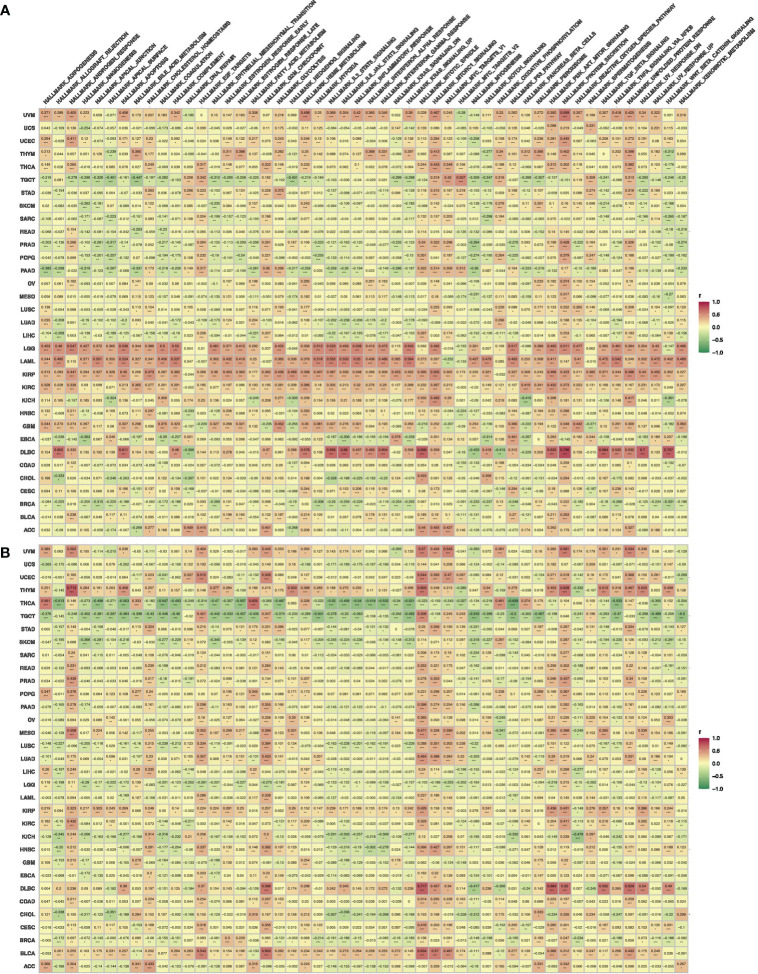
The correlations of HallMark pathways with ADIPOR1 **(A)** and ADIPOR2 **(B)**. *P < 0.05, **P < 0.01, ***P < 0.001.

The functional enrichment analysis showed that both ADIPOR1 and ADIPOR2 were associated with AMPK signaling, adiponectin-activated signaling pathway, vesicle transport, and lipid and glucose metabolism (**Figures 8A, B**). ADIPOR1 also exhibited a correlation with protein phosphorylation and dephosphorylation process, whereas ADIPOR2 exhibited a stronger correlation with protein ubiquitination and autophagy. The results from CancerSEA database revealed that ADIPOR1 was associated with quiescence, differentiation, DNA damage, and DNA repair (**Figure 8C**), and ADIPOR2 was associated with DNA damage, DNA repair, and angiogenesis (**Figure 8D**). The correlation analysis of hallmark pathways showed that ADIPOR1 was mainly correlated with HEME_METABOLISM, MTORC1_SIGNALING, PROTEIN_SECRETION, and UNFOLDED_PROTEIN_RESPONSE, and ADIPOR2 was mainly correlated with ANDROGEN_RESPONSE, MITOTIC_SPINDLE, E2F_TARGET, G2M_CHECKPOINT, MITOTIC_SPINDLE, MYOGENESIS, and PROTEIN_SECRETION (**Figure 9**).

### Drug sensitivity

**Figure 10:**
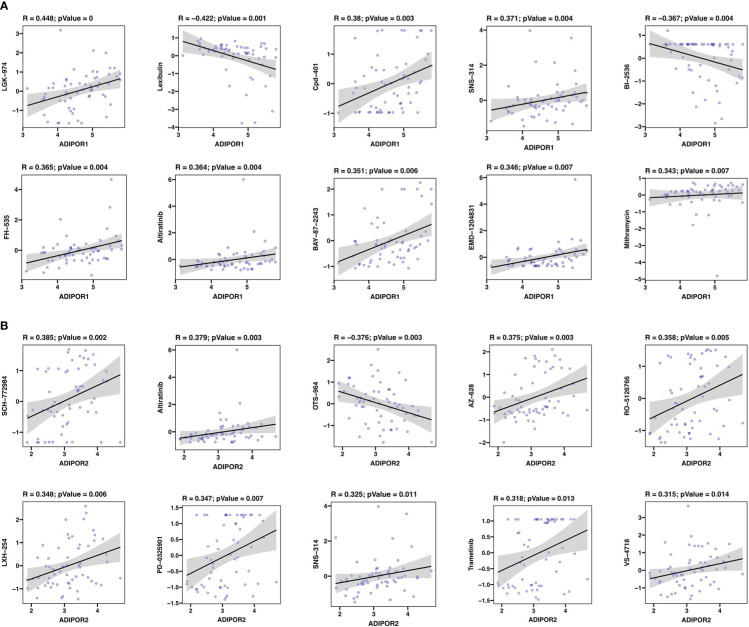
Drug sensitivity analysis. **(A)** The correlation between ADIPOR1 and drug sensitivity. **(B)** The correlation between ADIPOR2 and drug sensitivity.

Totally, ADIPOR1 was associated with 36 drugs, and ADIPOR2 was associated with 43 drugs. The top 10 drugs with highest significance were presented in **Figure 10**. LGK-974 was the drug mostly correlated with ADIPOR1, followed by Lexibulin and Cpd-401 (**Figure 10A**). SCH-772984 was the drug mostly correlated with ADIPOR2, followed by Altiratinib and OTS-964 (**Figure 10B**).

## Discussion

AdipoR1 and AdipoR2, encoded by ADIPOR1 and ADIPOR2 respectively, are the receptors of adiponectin. The activation of AdipoR1 and AdipoR2 by adiponectin result in the initiation of a series of signaling pathways, including AMPK and MAPKp38 pathways. Besides, they also function as a form of ceramidase activity to reduce the intracellular level of ceramide, which are linked to inflammation and cell death (3). Considering the complex biological functions of AdipoR1 and AdiopoR2, there is an urgent need to thoroughly analyze their contribution in cancer and anti-cancer immunity. Here, we conducted a pan-cancer analysis to investigate the roles of AdipoR1 and AdiopoR2 across cancers.

Although they both serve as the receptors of adiponectin, AdipoR1 and AdipoR2 possess different tissue specificities, indicating that they focus on different functional objectives. According to the THPA database, AdipoR1 was abundant in bone marrow, whereas AdipoR2 was abundant in white matter. A study from Korean found that ADIPOR1 rs16850799 and rs34010966 polymorphisms are correlated with bone mineral density in postmenopausal women (14). In the aged 5XFAD mouse model of Alzheimer’s disease, AdipoR2 was observed highly expressed in activated astrocytes (15). Our investigation found that the expression of ADIPOR1/2 was dysregulated in a large number of tumors, which is consistent with the findings of earlier studies showing ADIPOR1 and ADIPOR2 were implicated in various malignancies. Although they possessed significant correlations across cancers, the expression patterns of ADIPOR1 and ADIPOR2 were varied. ADIPOR1 was predominantly upregulated in cancers, whereas ADIPOR2 was downregulated in many cancers. In addition, the protein abundance of ADIPOR1/2 was observed dysregulated in many cancers as well. However, among cancers, the frequency of ADIPOR1/2 genetic alterations was low.

Some studies have focused on how ADIPOR1 and ADIPOR2 affect cancer prognosis. According to a study based on 369 patients, the overexpression of ADIPOR1 is related to lower overall survival outcomes in colorectal cancer (16). According to a study involving 866 patients, ADIPOR2 was related to lethal prostate cancer (17). In this study, we discovered that the expression levels of ADIPOR1/2 were linked to some clinicopathologic features, including OS and DFS. According to the results of univariate cox analysis and Kaplan-Meier plot analysis, ADIPOR1 was identified as a risky factor in ACC and LGG, and its overexpression was associated with their OS and DFS; ADIPOR2 was identified as a favorable factor in KIRC and a risky factor in MESO. Jehonathan H Pinthus et al. also reported that KIRC with metastasis expressed lower ADIPOR.

The aberrant epigenetic modulation is one of common characteristics in cancer. The current study indicated that the promoter DNA methylation levels of ADIPOR1/2 were rarely dysregulated in most cancers. Besides, they only exhibited slight correlations with 4 DNA methyltransferases. However, there were substantial correlations between ADIPOR1/2 and m6A enzymes. Especially in DLBC, they both exhibited positive associations with almost all the m6A readers, writers, and erasers.

Recently, ADIPOR1 and ADIPOR2 were discovered to be strongly linked to metabolic and immunological homeostasis in colorectal cancer (18). Therefore, we performed a comprehensive analysis to determine the correlation between ADIPOR1/2 and the tumor immune microenvironment. In General, immune cells express more ADIPOR1 than ADIPOR2. Compared to other immune cells, neutrophils were shown to express ADIPOR1 at a high level. It was reported that porcine ADIPOR1 transgenic mice exhibited higher expression levels of neutrophil chemokines, which indicated that ADIPOR1 owned a potential correlation with the recruitment of neutrophils (19). A study explored the distribution of ADIPOR1/2 on human peripheral blood mononuclear cells using flow cytometry. They found that, approximately, ADIPOR1 was present on 1% of T cells, 21% of NK cells, 47% of B cells, and 93% monocytes, and ADIPOR2 also exhibited similar distribution (20). ADIPOR1/2 also exhibited a correlation with the infiltration of immune cells. Our study revealed that both ADIPOR1 and ADIPOR2 were positively associated with CD4+ T cell and negatively associated with NK cell in most cancers. Qian Zhang et al. have confirmed that AdipoR1-deficent CD4+ T cells expressed lower Hypoxia-Inducible Factor-1α, and consequently was is suppressed to differentiate into Th17 cells because of the glycolysis inhibition (11). Additionally, ADIPOR2 exhibited a positive correlation with endothelial cells in many cancers. Angiogenesis is required for the progression of tumors, and endothelial cells are linked to the tumor metastasis and the formation of cancer-associated fibroblasts (21). ADIPOR1 and ADIPOR2 were also found differentially expressed in immune subtypes. In many cancers, subtype C4 (lymphocyte depleted) expressed higher ADIPOR1. C4 showed Th1 suppressed and a high M2 response (22). In KIRC and LGG, subtype C5 (immunologically quiet) expressed significantly high ADIPOR2. C5 displayed the lowest lymphocyte and highest macrophage in comparison to other immune subtypes (22).

ADIPOR1 and ADIPOR2 were correlated with stemness in many cancers, even though they only had weak correlations with TMB and MSI. It has been established that ADIPOR1 protects neural stem cells. J Song et al. found that AdipoR1 shielded neural cells against hyperglycemia both *in vivo* and *in vitro* (23). Nevertheless, more research is needed to fully understand the connections between ADIPOR1/2 and cancer stem cells. Compared to ADIPOR1, ADIPOR2 exhibited a stronger correlation with immunotherapy. In GSE106128, a DCs treated cohort, the expression of ADIPOR2 was upregulated after therapy. In GSE91061, an anti-PD-1 cohort, the patients with lower ADIPOR2 expression had better results. In addition, both the ADIPOR1 and ADIPOR2 exhibited extensive correlations with immune checkpoint genes in the majority of cancers, indicating that they may be used as predictors or novel targets of immunotherapy. CD274, encoding PD-L1, was observed significantly correlated with ADIPOR1 and ADIPOR2 in almost all the cancers. As a result, targeting ADIPOR1/2 is a viable tactic to increase the efficacy of anti-PD-L1. Besides, NRP1 also exhibited a strong correlation with ADIPOR1/2. As an immune memory checkpoint, NRP1 is capable of limiting long-term antitumor immunity (24). NRP1 not only promotes Treg activation, but also hinders CD8+ T cell responsiveness to immune checkpoint inhibitors (25).

Then, we investigated the function of ADIPOR1 and ADIPOR2 using bioinformatic methods. They possess certain distinctive features as well as some biological roles in common. ADIPOR1 exhibited potential correlations with phosphate metabolic process and regulation of dephosphorylation, whereas ADIPOR2 displayed potential correlations with protein ubiquitination and autophagy. CancerSEA database makes it possible to analyze the functions of gene at the single cell level. ADIPOR1 was found correlated with Quiescence status. Quiescent cancer cells are resistant to chemotherapy (13). Adiponectin-AdipoR1 axis enhances sensitivity to tyrosine kinase inhibitor sunitinib in metastatic renal cell carcinoma *via* inhibiting PI3K/AKT/NF-κB signaling (26). ADIPOR2 was correlated with angiogenesis, DNA damage, and DNA repair. Jennifer R Ridar et al. found that ADIPOR2 was positively associated with two measures of angiogenesis in lethal prostate cancer *via* immunohistochemistry (17). We also conducted a correlation analysis between ADIPOR1/2 and drug sensitivity. ADIPPOR1 exhibited a significant correlation with LGK-974, a small-molecule Porcupine (PORCN) inhibitor. PORCN is required for the Wnt ligand secretion, and the inhibition of PORCN by LGK-974 is a promising strategy to target Wnt-driven cancers (27). ADIPOR2 was strongly correlated with SCH-772984, an ERK inhibitor. Laura Broutier et al. identified the SCH-772984 as a potential drug for primary liver cancer through organoid culture system (28).

Of course, we have to acknowledge that there are some limitations in this study. Although we conducted a comprehensive analysis for the diverse roles of ADIPOR1 and ADIPOR2 across cancers, further experimental evidences are required to identify our results. Only a few tumor’s ADIPOR1/2 protein data are available in the public databases. The protein levels of ADIPOR1/2 in most cancers need to be validated by experiments. In certain malignancies, ADIPOR1 and ADIPOR2 performed advantageous roles; in other tumors, they played detrimental roles. Therefore, further investigation is needed into the roles played by ADIPOR1/2 in the particular malignancy.

## Conclusion

Although obesity has been associated with various tumors, it is still unclear how adipose tissues interact with malignant tissues. As receptors of endocrine factors secreted by adipose, AdipoR1/2 proteins (encoded by ADIPOR1/2 genes) are potential bridges between adipose tissues and tumors. To investigate the functions of ADIPOR1 and ADIPOR2 genes in tumors, we carried out a thorough pan-cancer analysis. Our research revealed that ADIPOR1 and ADIPOR2 are dysregulated in a wide range of tumors, and that they play critical roles in anti-tumor immunity as well as drug sensitivity, which indicated that adipose tissues might affect tumor tissues *via* interacting with AdipoR1/2 receptors. In all, AdipoR1/2 show capacities of prognostication in many tumors, and might serve as the potential biomarkers and drug targets in malignancies.

## Data availability statement

The original contributions presented in the study are included in the article/supplementary material. Further inquiries can be directed to the corresponding author.

## Author contributions

BY, ZC, and HY designed this study. BY, ZC, HY, YR, ZY, JH, CL, and YX conducted this study, including data collection, data analysis, and manuscript writing. All authors contributed to the article and approved the submitted version.
